# The impact of primary care reform on health system performance in Canada: a systematic review

**DOI:** 10.1186/s12913-016-1571-7

**Published:** 2016-07-30

**Authors:** Renee Carter, Bruno Riverin, Jean-Frédéric Levesque, Geneviève Gariepy, Amélie Quesnel-Vallée

**Affiliations:** 1 Department of Epidemiology, Biostatistics and Occupational Health, McGill University, 1020 Pine Avenue West, Montreal, QC Canada; 2 Department of Sociology, McGill University, 855 Sherbrooke Street West, Montreal, QC Canada; 3 Bureau of Health Information, Level 11 Sage Building, 67 Albert Avenue, Chatswood, NSW Australia; 4 Center for Primary Health Care and Equity, University of New South Wales, Level 3, AGSM Building, Sydney, Australia; 5 Institute for Health and Social Policy, McGill University, 1130 Pine Avenue West, Montreal, QC Canada

**Keywords:** Primary care reform, Payment models, Team-based practice, Canada

## Abstract

**Background:**

We aimed to synthesize the evidence of a causal effect and draw inferences about whether Canadian primary care reforms improved health system performance based on measures of health service utilization, processes of care, and physician productivity.

**Methods:**

We searched the Embase, PubMed and Web of Science databases for records from 2000 to September 2015. We based our risk of bias assessment on the Grading of Recommendations Assessment, Development and Evaluation guidelines. Full-text studies were synthesized and organized according to the three outcome categories: health service utilization, processes of care, and physician costs and productivity.

**Results:**

We found moderate quality evidence that team-based models of care led to reductions in emergency department use, but the evidence was mixed for hospital admissions. We also found low quality evidence that team-based models, blended capitation models and pay-for-performance incentives led to small and sometimes non-significant improvements in processes of care. Studies examining new payment models on physician costs and productivity were of high methodological quality and provided a coherent body of evidence assessing enhanced fee-for-service and blended capitation payment models.

**Conclusion:**

A small number of studies suggested that team-based models contributed to reductions in emergency department use in Quebec and Alberta. Regarding processes of diabetes care, studies found higher rates of testing for blood glucose levels, retinopathy and cholesterol in Alberta’s team-based primary care model and in practices eligible for pay-for-performance incentives in Ontario. However pay-for-performance in Ontario was found to have null to moderate effects on other prevention and screening activities. Although blended capitation payment in Ontario contributed to decreases in the number of services delivered and patients seen per day, the number of enrolled patients and number of days worked in a year was similar to that of enhanced fee-for-service practices.

## Background

Between 2000 and 2006 the Primary Health Care Transition Fund (PHCTF) contributed $800 million towards reforming primary care in Canadian provinces and territories. This effort was aimed to address the health service needs of the country’s ageing population and growing burden of chronic disease [1]. Common objectives were in keeping with the idea of the patient-centered medical home (PCMH): increase access to primary care, promote multidisciplinary team-based care, and improve chronic disease management [2].

The reform initiatives implemented across Canadian jurisdictions have largely emphasized quality improvement and incentive-base levers, and/or organizational changes to practice, including the formation of primary care teams, partnerships, networks, or federations of physicians [3]. In addition, there is growing recognition that the mechanism of physician remuneration plays a role in upholding primary health care objectives such as continuity and quality of care [4, 5]. Organizational change to primary care practice in Canada is understood to encompass both team-based service delivery involving allied health professionals, and new blended payment models that seek to promote patient enrolment, continuity, and coordination of care.

Syntheses of primary care reforms in Canada have called for rigorous evaluation of reforms using appropriate health system performance indicators [1, 3]. Although a number of studies on various aspects of provincial reforms have been published, no systematic review of the literature exists on the effects produced from practice-level organizational changes [6]. Given that models are evolving and new ones are being proposed, it is imperative to synthesize the knowledge accumulated on these reforms to support future policies. We sought to systematically review and assess the published and peer-reviewed literature that describes practice-level organizational reforms in Canada introduced during or after the PHCTF. We specifically aimed to synthesize the evidence of effects and draw inferences about whether Canadian primary care reforms improved health system performance based on measures of health service utilization, processes of care, and physician productivity.

## Methods

### Data sources and inclusion criteria

Our review focused on organizational reforms to primary care in Canada, namely, the formation of group practices (including team-based practices), new payment models intended to support group practice, or both. Provinces that introduced quality and incentive-based reforms without an emphasis on providing financial support for group or team-based practices were not included in this review. Further, those that did implement organizational reforms to practice but only did so in pilot projects were also excluded. We limited the scope of our review to Alberta, Ontario and Quebec where system-wide reform initiatives that meet the above criteria have been pursued [3].

In Alberta and Quebec, the team element of including nurses and other health professionals in primary care practice was an integral feature of the Primary Care Network (PCN) and Family Medicine Group (FMG) reforms. For this reason, we refer to these practice models as ‘teams’ throughout the text and in the results tables. However, we refer to the more general term of ‘group practice’ in Ontario to underscore the fact that payment reforms were the main changes that were followed by the creation of Family Health Teams that operate within specific new payment models. To elaborate, only physicians that accept a blended capitation payment and are members of Family Health Organizations (FHO) or Family Health Networks (FHN) can become a Family Health Team (FHT). As such, while a group of physicians may practice in the same FHO or FHN, they are not necessarily an FHT. In instances where FHTs are no specifically referenced, we used the term ‘group practice’ when discussing reforms in Ontario.

We searched the Embase, PubMed and Web of Science databases for records from 2000 to September 2015. We used both general search terms that referred to primary care reform in Canada and search terms specific to the provinces of interest (Appendix A). Studies were eligible if they sought to draw inferences on the effects of new organizational or payment models in the Canadian provinces of interest. Search terms were modified according to the database, where appropriate. We also conducted a hand-search of references cited in the studies included in the review.

### Study selection

Two reviewers (RC and BDR) independently screened titles and abstracts of records identified from the database search and included those based on the following criteria: (1) the study pertained to Canada; (2) the study examined reforms in Alberta, Quebec, or Ontario; and (3) the study reported quantitative measures of effect. Studies were excluded if the intervention could not be classified under the defined categories, if the outcome did not fall into the categories outlined in Table 1, or if the study was a commentary. The same two reviewers further investigated studies eligible for inclusion as full text.

**Table 1:** Outcome assessment for systematic review

Outcome	Examples
Health service utilization	Visits to the emergency department Hospital admissions Visits to specialists
Processes of care	Delivery of guideline recommended chronic disease management Delivery of clinical preventive services
Physician costs/productivity	Number of services delivered Number of patients seen Risk selection of patients

We synthesized and organized the results of the full-text studies included in the review according to the three outcome categories: health service utilization, processes of care, and physician costs and productivity. We reported estimates of adjusted measures of effect and precision. We did not summarize results from studies that did not provide adjusted measures of association. Our descriptive synthesis also excluded cross-sectional studies. Although cross-sectional studies can be used to detect associations between factors, they cannot provide evidence for the effect of interventions because temporality cannot be established. Considerable heterogeneity between studies with regard to interventions and measures of outcome precluded a meta-analysis. Our review follows the Preferred Reporting Items for Systematic Reviews and Meta-Analyses (PRISMA) statement [7].

### Data extraction and quality assessment

Two reviewers (RC and BDR) independently extracted data and performed a risk of bias assessment for each full-text study included in the review. We first piloted our extraction and quality assessment forms on four studies to standardize our approach. We based our risk of bias assessment on GRADE guidelines [8] with adaptations to account for issues of selection bias and exposure definitions specific to our context. Although randomized controlled trials are considered the gold standard for evaluating interventions, it is widely acknowledged that this study design is not feasible for evaluating certain exposures. Other study designs, such as before and after studies with a control group and interrupted time series analyses, are recognized as having the potential to contribute high quality evidence [9]. Studies that exploited a natural experiment setting by including cohorts of physicians or patients before and after the introduction of a reform and also incorporated a control group were granted a higher rating. There were two main reasons for this: (1) accounting for pre-intervention trends controls for observed and unobserved time fixed confounding factors, and (2) the addition of the control group addresses secular trends in the outcome (i.e. factors association with changes in the outcome that vary over time), assuming the control group serves as an appropriate counterfactual for what would have occurred to those that experienced the intervention if they had not been exposed to it.

Since physician and patient participation in new primary care models is voluntary, studies that addressed the underlying mechanisms determining individual membership in the intervention or control groups either by design or analytical approach were rated favorably. By comparison, a study that did not provide a clear definition of the intervention or control groups received a lower rating due to a greater risk of bias. The body of evidence for each outcome was then summarized according to GRADE categories of high, medium, or low (Appendix B) [10]. We held reconciliation meetings to compare the information extracted from each study and each reviewer’s evaluation of study quality. A third party arbitrator (AQV) was available in the event that disagreements between the two reviewers could not be resolved (a situation that did not arise).

## Results

### Search results and study characteristics

**Fig. 1:**
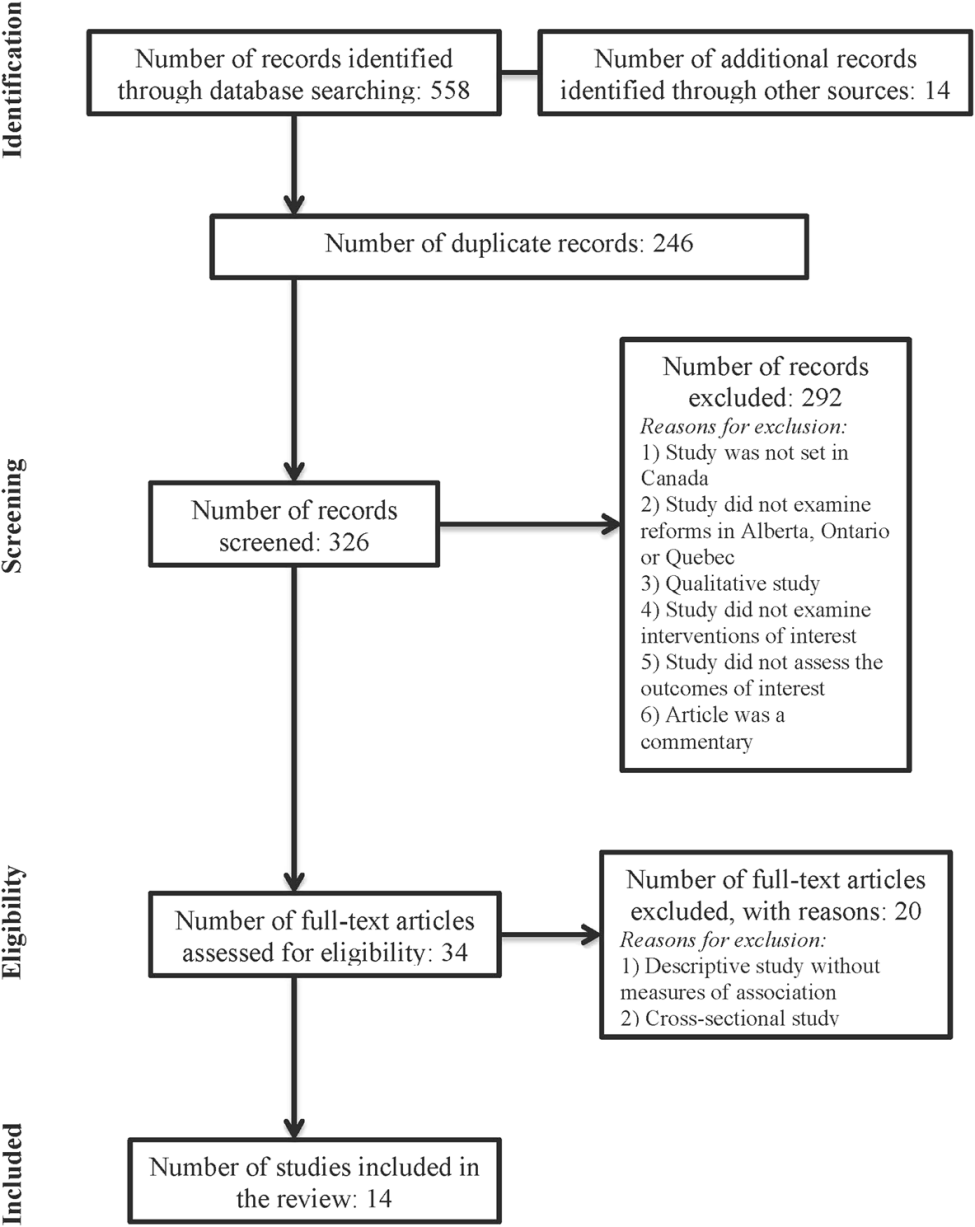
PRISMA flow diagram

Our search identified 572 studies, 558 of which were from the databases and 14 from a hand search. After removing the duplicate records, we screened 326 studies and excluded 292 based on the reasons listed in Fig. 1. In a full-text review, we then assessed the 34 remaining studies for eligibility and excluded 20 for reasons also cited in Fig. 1. Data extraction and a risk of bias assessment were performed for the 14 studies included in the systematic review. Of the 14 studies included, the majority focused on the effects of new payment models in Ontario [11–19] (Table 2). The remaining 5 studies from Quebec and Alberta focused on the effects of team-based aspects of primary care reforms. [20–24] The publication dates spanned from 2009 to 2015. In 8 of the studies, specific sub-populations were studied all of which were comprised of individuals with chronic disease. These included 4 studies on patients with diabetes [13–15, 20], and 4 on chronic conditions in general [17, 21, 23, 24]. With regard to primary outcomes of interest, 3 studies focused on health service utilization [20–22], 6 on processes of care [11–15, 23, 24], and 4 on physician costs and productivity [16–19] (Table 2). Of the 14 studies, 2 assessed independent associations between several predictors (including primary care reform models) and health system performance outcomes, and 12 specifically assessed the causal effect of defined interventions compared to a control group.

**Table 2:** Characteristics of studies included in the systematic review

Organizational change	Study				Population			Intervention			
	Source	Year	Design	Province	Study population	*N*	Sub-population	Study follow-up	Intervention^a^	Comparison group	Primary Outcome
Team-based primary care models	Héroux, J. et al.	2014	Cohort study	Quebec	Patients	231,938	Vulnerable patients	3 years	Family medicine groups	Individuals not enrolled in a Family medicine group	Health service utilization
	Lévesque, J.F. et al.	2012	Cohort study	Quebec	Patients	598	Chronically ill (diabetes, heart failure, COPD, arthritis)	18 months	Family medicine groups^b^	Individuals receiving care in community health centers	Processes of care
	Feldman, D.E. et al.	2012	Cohort study	Quebec	Patients	598	Chronically ill (diabetes, heart failure, COPD, arthritis)	18 months	Family medicine groups^b^	Individuals receiving care in community health centers	Processes of care
	Manns, B.J. et al.	2012	Cohort study	Alberta	Patients	154,928	Diabetes	1 year	Primary care networks	Individuals not enrolled in a Primary Care Network	Health service utilization
	Campbell, D.J.T. et al.	2012	Cohort study	Alberta	Patients	106,653	Diabetes Low-income First Nations	1 year	Primary care networks	Individuals in the sub-population of interest not enrolled with a Primary Care Network	Health service utilization
Payment models and incentives	Kiran, T. et al.	2014	Before and after	Ontario	Patients	Cervical cancer: 3,056,337 Breast cancer: 1,600,645 Colorectal cancer: 3,713,963	NA	10 years	Pay for performance	Outcome measures in the pre-intervention period	Processes of care
	Li, J. et al.	2014	Before and after	Ontario	Physicians	2,154	NA	10 years	Pay for performance	FFS	Processes of care
	Kantarevic, J. et al.	2013	Before and after	Ontario	Physicians	3,588	Diabetes	2 years	Blended capitation	Enhanced FFS	Processes of care
	Kiran, T. et al.	2012	Cohort study	Ontario	Patients	58, 927	Diabetes	5 years	Payment models^c^	Outcome measures in the pre-intervention period	Processes of care
	Jaakimainen, L.R. et al.	2011	Before and after	Ontario	Physicians	3,940	NA	4 years	Payment models^d^	Outcome measures in the pre-intervention period	Processes of care
	Kantarevic, J. et al.	2015	Before and after	Ontario	Physicians	3,428	NA	7 years	Blended capitation	Enhanced FFS	Physician costs/productivity
	Kantarevic, J. et al.	2014	Before and after	Ontario	Physicians	673	Complex and vulnerable patients	2 years	Capitated incentive payment	Enhanced FFS	Physician costs/productivity
	Kralj, B. et al.	2013	Before and after	Ontario	Physicians	4,156	NA	4 years	Blended capitation	Enhanced FFS	Physician costs/productivity
	Kantarevic, J. et al.	2011	Before and after	Ontario	Physicians	7,003	NA	17 years	Enhanced FFS^e^	FFS	Physician costs/productivity

^a^Where the name of the practice model was not specified, only the payment modality is listed. Accordingly: Harmonized (blended capitation) models include: Family Health Networks and Family Health Organizations, Non-harmonized (enhanced FFS) models include: Family Health Group and Chronic Care Model

^b^The control group was identified according to how the comparisons were being made in the article and by what was listed as the reference category in a results table from a regression model

^c^The intervention group was identified as ‘payment models’ in instances where studies from Ontario survey all payment models as opposed to studying the effect of a single payment model in relation to a control group. Where the name of the practice model is given, we also specified the payment modality associated with it

^d^The authors examined the Family Health Group model (enhanced FFS) and the Family Health Network model (blended capitation). Enhanced FFS refers to predominantly FFS payment with bonuses for extended opening hours and patient enrolment

^e^The authors examined the Family Health Group model (enhanced FFS)

### Effectiveness of interventions

#### Health service utilization (Table 3)

**Table 3:**
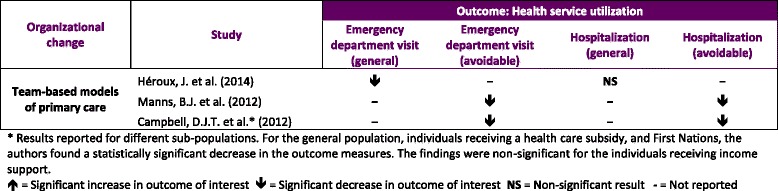
Results for health service utilization outcome

Three studies examined health service utilization as a primary outcome focusing on team-based aspects of reforms among chronically ill or elderly patients in Quebec [21] and Alberta [20, 22]. Although the observed tendency pointed to statistically significant decreases in ED visits, the evidence on admissions was mixed. Héroux et al. [21] found a decrease in the rate of visits to the ED attributed to Family Medicine Group (FMG) enrolment within a vulnerable group of patients defined by chronic disease or older age (RR 0.93; 95 % CI 0.90, 0.95), yet null effects on hospital admissions (RR 1.02; 95 % CI 0.98, 1.06). In studies of the effects of Primary Care Networks (PCNs), Manns et al. [20] reported an 18 % reduction in the rate of avoidable ED visits made by patients with diabetes affiliated with PCNs relative to those in non-PCNs (RR 0.82; 95 % CI 0.76, 0.88) and a 19 % reduction in the rate of avoidable admissions for the same patient group (RR 0.81; 95 % CI 0.75, 0.87). Campbell et al. [22] also found significant reductions in the rate of avoidable use of the ED and admissions within the general population (RR 0.75 95 % CI 0.67, 0.85), low-income population (RR 0.71; 95 % CI 0.54, 0.94), and First Nations (RR 0.74; 95 % CI 0.59, 0.93).

#### Processes of care (Tables 4 and 5)

**Table 4:**
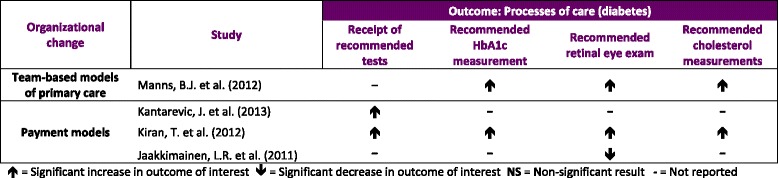
Results for processes of care outcome (diabetes)

**Table 5:**
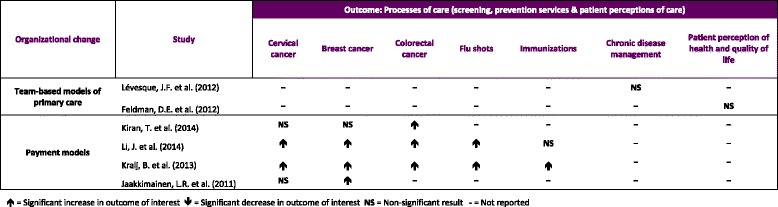
Results for processes of care outcome (screening, prevention services and patient perception of care)

Three studies examined measures related to the process of care for patients with diabetes. These consisted of studies examining payment reforms in Ontario:1 pre/post study with a control group [13], 1 pre/post study with no control group [15], and 1 cohort study [14]. There was 1 cohort study on team-based practices in Alberta where processes of diabetes care were secondary outcome measures [20, 22]. Manns et al. [20] found that relative to patients with diabetes not enrolled in a PCN, the rate of patients receiving blood glucose monitoring was 2 % higher than non-enrolled patients (RR 1.02; 95 % CI 1.01, 1.03). For the same comparison, the rate of visits to the ophthalmologist was 19 % higher (RR 1.19; 95 % CI 1.17, 1.21). The authors also found that relative to non-PCN patients, the rate of cholesterol measurement was 3 times greater (RR 1.03; 95 % CI 1.02, 1.04). Both pre/post studies assessed the effect of payment reforms in Ontario on diabetes processes of care. Jaakimainen et al. [15] estimated 15 % and 14 % decreases in the proportion of patients with diabetes receiving an annual eye exam after enrolling with physicians receiving blended capitation and enhanced FFS payments, respectively. Kiran et al. [14] found greater adherence to recommended testing for diabetes patients in the years following the introduction of an incentive fee code (RR 1.22; 95 % CI 1.21, 1.23) however the increases in trend were already occurring prior to introducing the incentive. Using a difference-in-differences analysis, Kantarevic et al. [13] studied physician participation in the Diabetes Management Initiative, another P4P scheme designed to incentivize adherence to guidelines. They found that patients with diabetes receiving recommended tests in Family Health Organizations (FHO) were 8 % more likely to receive recommended tests relative to those in the Family Health Group (FHG) enhanced FFS model.

All four studies examining various outcomes related to screening and prevention activities in new payment models used pre/post study designs [11, 12, 15, 18]. Two of these studies included a control group using a difference-in-differences analysis [12, 18]. Kralj et al. [18] examined the effect of blended capitation payment on the delivery of the cancer screening and preventive care targets (senior flu shots, Pap smears, mammograms, immunizations and colorectal cancer screening). Their findings showed that compared to physicians in the FHG model who were paid by enhanced FFS, those in the blended capitation FHO model were 7 to 11 % more likely to meet preventive care quality targets. Two studies [11, 12] examined the effect of a P4P incentive on the delivery of the same cancer screening and preventive care targets with results indicating null to moderate effects. Li et al. [12] measured the effect of P4P in new patient enrolment models. Relative to physicians in traditional FFS practices, results indicated statistically significant increases of 2.8, 4.1, 1.8 and 8.5 percentage points in the delivery of senior flu shots, Pap smears, mammograms and colorectal cancer screening, respectively. Using an interrupted time-series design, Kiran et al. [11] found a statistically significant increase of 4.7 % in the rate of colorectal cancer screening after the introduction of the P4P incentive. However, the results showed no statistically significant changes in the rates of breast and cervical cancer screening. Finally, Jaakimainen et al. [15] reported overall changes of less than 5 % in the proportion of women screened for cervical and breast cancer after joining an FHG or FHN. However, changes of over 5 % were reported for the proportion of individuals receiving any type of colorectal cancer screening.

Two studies from Quebec examined outcomes related to patient-reported health and chronic illness care in FMGs [23, 24]. In comparison to patients treated in community health centers, Levesque et al. reported a small but non-significant effect of FMGs on patient assessment of chronic illness care. Using the same comparison group, Feldman et al. found no significant effects of FMGs on improving patients’ physical health, mental health or health related quality of life.

#### Physician costs and productivity (Table 6)

**Table 6:**
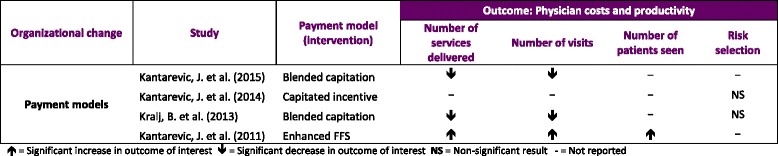
Results from physician costs and productivity outcome

All 4 studies analyzed the data using a difference-in-differences approach and propensity score matched physicians at baseline [16–19]. Kantarevic et al. [16] studied the differences in the number of patient visits and services delivered by physicians per day. Relative to enhanced FFS models, physicians paid by blended capitation experienced between 3.8 % and 4.2 % reduction in the number patient visits per day, and between 5 % and 6 % reduction in the number of services delivered per day. Kralj et al. found similar reductions of 6 % for the number of visits per day and 7 % for the number of services delivered per day [18]. In an earlier study, Kantarevic et al. [19] examined similar outcomes comparing physicians in enhanced FFS models with those in traditional FFS models. Relative to physicians in traditional FFS practices, those paid by enhanced FFS increased the number of patient visits by 6.3 % and the number of services by 9.3 %. Kantarevic et al. [17] also addressed the question of cost-shifting and risk selection in blended capitation models following the introduction of a new incentive payment to enroll complex and vulnerable patients. The results revealed no statistically significant changes in physician behavior relative to those practicing in traditional FFS models that were not eligible for the incentive payment. Kralj et al. [18] also found non-significant results regarding risk selection when comparing physicians in blended capitation practices with those in enhanced FFS practices.

### Quality of evidence assessment

GRADE guidelines identify 4 main components for assessing the quality of the body of evidence: study design, risk of bias, inconsistency and indirectness of results [25]. All of the studies included in the review were observational. The risk of bias varied by outcome categories (Table 7), primarily due to whether the methodology accounted for selection bias and whether clear definitions of intervention and control groups were provided. Several studies relied on administrative population-based data allowing for increased statistical power to detect effects. In 7 studies, large longitudinal databases facilitated the use of propensity scores to create matched samples of patients or physicians at baseline prior to the introduction of an intervention. This approach aimed to address the selection of physicians and patients into new primary care models.

**Table 7:** Quality of evidence assessment

Outcome	Reform	Number of studies	Study design	Risk of bias	Directness	Consistency	Overall assessment of the evidence
Health service utilization	Team-based models	3	Cohort studies	No risk of serious bias	Serious indirectness^a^	No serious inconsistency	Moderate
Process of care	Team-based models	3	Cohort studies	Risk of serious bias	Serious indirectness^a^	Serious inconsistency	Low
	Payment models	6	Before and after and cohort studies	Risk of serious bias	Serious indirectness^b^	Serious inconsistency	Low
Physicians costs and productivity	Payment models	4	Before and after	No risk of serious bias	No serious indirectness	No serious inconsistency	High

^a^The main source of indirectness stems from the evaluation of different team-based interventions. Given the small number of studies, we conducted a pooled assessment of the evidence from Alberta and Quebec in order to provide an overall assessment of the evidence

^b^The main source of indirectness stems from results on a number of different interventions examined in relation to payment models in Ontario that we pooled in order to provide an overall assessment of the evidence

Variations within team-based or payment-based reform initiatives across Canadian jurisdictions and across included studies resulted in indirect comparisons, which decreased the overall quality. Despite this, studies on team-based reforms from Alberta and Quebec provided consistent evidence of reductions in emergency department use, which resulted in a moderate quality rating. For processes of care, risk of serious bias and evidence of indirectness in outcome measures and interventions led to low quality evidence. The methodologies and analytical approaches used in studies examining physician productivity outcome measures presented no risk of serious bias and no serious indirectness or inconsistencies in their comparisons. We therefore judged the overall quality of the evidence as high for this outcome.

## Discussion

Our review provides the first systematic evidence synthesis of the literature on the effects of recent organizational changes to primary care in Canada on health system performance outcomes. We found moderate quality evidence that interdisciplinary team-based models of care such as Quebec’s FMGs and Alberta’s PCNs led to reductions in emergency department use, but the evidence was mixed for hospital admissions. We also found low quality evidence that team-based models, blended capitation models and pay-for-performance incentives led to small and sometimes non-significant improvements in processes of care as measured by the delivery of screening and prevention services and chronic disease management. Studies examining the effects of new payment models in Ontario on physician costs and productivity were of high methodological quality and provided a coherent body of evidence assessing enhanced FFS and blended capitation payment models. Findings indicated that moving from enhanced FFS to blended capitation reduced the number of patients seen per day yet the number of enrolled patients and days worked per year remained the same.

Our findings on new payment models in Ontario align with economic theory that FFS incentivizes increasing the volume of services while blended capitation tends to produce reductions. A blended capitation model may be more efficient than FFS if quality of care is maintained or elevated and the delivery of inappropriate services is reduced [26]. Also, similar to findings from other systematic reviews, we found that pay-for-performance incentives yielded some benefits but the evidence was inconsistent across outcomes, suggesting that these types of interventions must be carefully designed and executed. This is particularly relevant to the case of Ontario in which blended payment models make up the core of the primary care reforms and where P4P became available to physicians paid through blended capitation. In the discussion of their findings on P4P for diabetes management, Kantarevic et al. [13] make reference to both the incentive amount and the level of cost-sharing borne by physicians (i.e. after receiving a fixed payment, the level of reimbursement received by physicians for delivering incremental services, whether these be outside of a defined basket services or when the cost of treating a patient exceeds the fixed payment amount) as important factors to consider when assessing the effectiveness of P4P incentives in striking a balance between the quality and quantity of services delivered to patients. Accordingly, the inconclusive evidence regarding physician responses to P4P incentives are likely due to differences study methodologies and/or compensation schemes. For this reason, incentive payments should be designed with the overarching payment model in mind [13].

Our review also indicated discrepancies in findings across studies that may be attributable to jurisdictional differences in implementation. Notably, findings from evaluations of Quebec’s FMGs are less convincing than those from evaluations of Alberta’s PCNs, although both are team-based primary care models adapted from the PCMH. In fact, a systematic review on the effects of the PCMH in the US highlighted that despite shared objectives of better coordination of care, increased access to and continuity of services, definitional frameworks and implementation approaches varied widely [27]. And indeed, a 2015 report from Quebec’s Auditor General highlighted the lack of a governmental framework for their implementation and evaluation, which may provide some insight into why FMGs are not performing as well as expected [28].

Our review also revealed gaps in the Canadian evidence of effects of primary care reforms. First, most longitudinal studies included in this review were performed with samples of chronically ill adults, while fewer were conducted in the general adult population and none were conducted in children. Few studies accounted for selection of physicians and patients into emergent reform initiatives by employing analytical techniques, such as difference-in-differences, propensity scores or instrumental variables. Future research should aim to evaluate indicators of health utilization, processes of care and physician productivity for which a change over time clearly shows improvements for patients and for the health system. Such indicators might include hospital readmissions and timely post-discharge follow-up care for targeted patient subgroups. Further, early adopters of reforms are generally more receptive to change [29], and an investigation into the dynamics of early versus late adopters could help gain insight into the potential for reforms to improve quality and performance.

Our results have implications for both policymakers and researchers. Firstly, we find evidence that interventions succeed in meeting health system objectives when they are targeted and carefully designed. For instance, although FMGs in Quebec were introduced with an aim to improve access to and quality of care for chronically ill patients, it is unclear whether this can be achieved when the establishment of chronic disease management programs is not integral to the reform. Where financial incentives such as pay-for-performance are concerned, consideration for the overarching physician payment model is central to designing a reward payment that avoids perverse incentives for patient risk selection by physicians. Secondly, the different nature of the interventions is of importance to researchers seeking to assess impacts of primary care reform. Unlike reforms to payment which can only be implemented in one way (eg. a physician is either paid via blended capitation or FFS), identifying effects is more difficult in team-based reforms since versions of the intervention differ by how each practice implements the model (eg. the extent to which nurses are integrated in patient case management). Finally, policy-makers and researchers should provide clear definitions of the roles, activities and processes enacted under the transformations intended by the reforms to best inform future efforts to enhance primary care.

### Limitations

In this review, we tried to minimize heterogeneity by including only population-wide interventions that occurred after 2000, and by restricting our criteria to studies with a longitudinal design. Nevertheless, considerable heterogeneity in interventions and in methodological approaches remained, which precluded meta-analysis and sub-group analysis. Although administrative data is useful for quantifying the change induced by the introduction of reforms, it is limited in terms of gauging the heterogeneity of reform implementation within practices. The effects of new models may be attenuated if high and low performing practices are not distinguishable from each other. Qualitative studies from Quebec and Ontario have described variation in how nurses are integrated into practice according to whether they hold responsibilities for patient care or are treated as assistants [30, 31]. Future reviews should consider the body of qualitative evidence to address normative questions that take stock of what is taking place within healthcare organizations versus what should be taking place.

## Conclusion

The quality of evidence ranged from low to high for each indicator of performance. Given our interest in isolating the causal effects of reforms we focused on methodologies that used an appropriate comparison group that controlled for factors contributing to the selection of physicians and patients into new primary care models and secular time trends in the outcome. The small number of studies from Alberta and Quebec suggested that team-based models contributed to reductions in ED use. Regarding processes of care, the evidence indicated that increases in preventive care services could be attributed to blended capitation models and P4P in Ontario. Although blended capitation appeared to lead to decreases in the number of services delivered and patients seen per day, the number of enrolled patients and number of days worked in a year was similar to that of enhanced FFS practices. Based on this review, we recommend methodologies that generate evidence on reform effects, particularly in Quebec and Alberta where only a small body of literature exists.

## Abbreviations

CI, confidence Interval; FFS, fee for service; FHG, family health group; FHO, family health organization; FMG, family medicine group; GRADE, grading of recommendations assessment, development and evaluation; P4P, pay for performance; PCMH, patient-centered medical home; PCN, primary care network; PHCTF, primary health care transition fund; PRISMA, preferred reporting items for systematic reviews and meta-analyses; RR, rate ratio

## Appendix A

Search strategy and terms

Keyword search terms:

Family Medicine Group*

Group de médecine familiale

Network clinic*

Integrated network clinic*

Clinique* réseau*

Clinique* réseau* intégrée*

Family Health Team*

Family Health Organization*

Family Health Group*

Primary Care Network*

Comprehensive Care Model*

Primary Health Care Initiative

Search strategies:

Canad* AND (“family medicine group*”)

Canad* AND (“integrated primary care network*”)

Canad* AND (“family health team*”)

Canad* AND (“family health organization*”)

Canad* AND (“family health group*”)

Canad* AND (“primary care network*”)

Canad* AND (“comprehensive care model*”)

Canad* AND (“enhanced fee for service”)

Canad* AND (“capitation”) AND (“payment”)

Canad* AND (“pay for performance”)

## Appendix B

**Table 8:** Quality rating categories according to GRADE guidelines for assessing the body of evidence

Quality rating	Definition
High	Confidence that the true effect is close to the estimated effect
Moderate	True effect is expected to be close to the estimated effect however it may be significantly different
Low	True effect may be very different from the estimated effect
